# Real-time single-pixel imaging using a system on a chip field-programmable gate array

**DOI:** 10.1038/s41598-022-18187-8

**Published:** 2022-08-18

**Authors:** Ikuo Hoshi, Tomoyoshi Shimobaba, Takashi Kakue, Tomoyoshi Ito

**Affiliations:** Graduate School of Engineering, Chiba-University, 1-33, Yayoi-cho, Inage-ku, Chiba, Japan

**Keywords:** Imaging and sensing, Other photonics, Electrical and electronic engineering

## Abstract

Unlike conventional imaging, the single-pixel imaging technique uses a single-element detector, which enables high sensitivity, broad wavelength, and noise robustness imaging. However, it has several challenges, particularly requiring extensive computations for image reconstruction with high image quality. Therefore, high-performance computers are required for real-time reconstruction with higher image quality. In this study, we developed a compact dedicated computer for single-pixel imaging using a system on a chip field-programmable gate array (FPGA), which enables real-time reconstruction at 40 frames per second with an image size of 128 × 128 pixels. An FPGA circuit was implemented with the proposed reconstruction algorithm to obtain higher image quality by introducing encoding mask pattern optimization. The dedicated computer can accelerate the reconstruction 10 times faster than a recent CPU. Because it is very compact compared with typical computers, it can expand the application of single-pixel imaging to the Internet of Things and outdoor applications.

## Introduction

Conventional imaging uses an image sensor, such as charged-coupled-device cameras. By contrast, single-pixel imaging is a unique technique that uses only a single-element detector, such as a photodiode^1^. In this technique, the target object image is reconstructed from acquired light intensities and known encoding mask patterns. Various reconstruction algorithms have been proposed for single pixel imaging, including correlation calculations called ghost imaging^2,3^, the Fourier transform and Hadamard transform-based methods^4,5^, optimization methods solving an ill-posed problem^6,7^, and deep learning^8–10^. Because single-element detectors are used in single-pixel imaging, unlike in the case of conventional imaging, it enables high sensitivity, broad wavelength, and noise robustness imaging. Single-pixel imaging can be applied to remote sensing^11^, three-dimensional imaging^12,13^, terahertz imaging^14,15^, cytometry^16^, and Internet of Things (IoT) cameras for use in surveillance, factories, hazardous dark areas, and outdoors, e.g. infrared camera^17^ and gas leak detection^18^.

Single-pixel imaging is computation intensive and requires compact and efficient devices for specific applications, e.g. IoT applications. Embedded computers can be the potential solution, but they are not suitable for the reconstruction calculation due to their low computational performance. Therefore, a small computer with high computational performance implemented in a single large-scale integration (LSI) chip is required. Dedicated computers for single-pixel imaging have been developed using field-programmable gate arrays (FPGAs). A FPGA is an LSI chip that can freely rewrite logic circuits on site. FPGAs can perform high-performance computation by designing application-specific circuits. There are several previous studies using FPGAs. Only the LED control was performed by an FPGA^19,20^, while the reconstruction computation was performed by a personal computer (PC). Iterative computation method, Hadamard transform, and differential ghost imaging (DGI) were implemented^21–23^, respectively, to speed up image reconstruction. The entire process from reconstruction calculation to display was performed on an FPGA^24^. However, the number of pixels and image quality, computation time, and image quality at low sampling are issues.

In this study, we developed a dedicated computer using a system on a chip (SoC) FPGA for single-pixel imaging. A SoC FPGA is an LSI in which an embedded CPU and FPGA are implemented on a single chip. It has higher computing performance than an alone embedded CPU, more flexibility than an alone FPGA, and can be much smaller than a computer. In addition, the selection of reconstruction algorithms to should be implemented as a computational circuit is important in designing a computer dedicated to single-pixel imaging. Although FPGAs have high computing performance, they have a limitation of hardware resources and are not good at complex calculations, such as division and square root calculations. Among algorithms, optimization methods solving an ill-posed problem and deep learning can obtain high quality reconstructions in single-pixel imaging^6–10^; however, deep learning has the problem of insufficient hardware resources, and optimization methods have the problem of computational load due to the iteration fashion.

Ghost imaging-based correlation calculation^2,3^ is suitable for FPGA implementation because of its low memory usage and the simplicity of calculation form. In this study, we used our ghost imaging algorithm, which introduces encoding mask patterns optimization^25^. It improves image quality with slight impact on the required computation and memory. In the proposed dedicated computer, the size of reconstructed images is 128 × 128 pixels, with up to 1,024 encoding mask patterns. We evaluate the dedicated computer in terms of the image quality and computational speed using frames per second (fps) in numerical and optical experiments. Finally, we demonstrate a real-time display system of single-pixel imaging using the dedicated computer. The dedicated computer we have developed can obtain higher image quality than references^19,20,22–24^ at the same sampling rate, a larger image size than references^19–21,23,24^ and faster speed than references^21–24^, enabling real-time display with both size, image quality and speed. Because the dedicated computer is extremely compact compared with typical computers, it can expand the application of single-pixel imaging to the IoT and outdoor applications. Specific applications using dedicated computers include implementation in satellite topographical surveying^26^, taking advantage of the small size and power-saving features of dedicated computers. It can also be used for object tracking^27–29^ to build a car navigation IoT system. In addition, dedicated computers can be used to reconstruct wide-wavelength images that require computing power^30^.

## Results

### Experimental system

**Figure 1:**
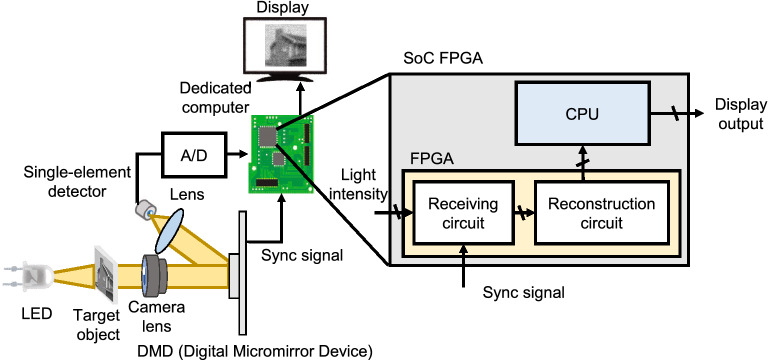
Schematic of the experimental setup. A camera lens forms the image of a target object on a DMD. The image of the target object is modulated by encoding mask patterns displayed on the DMD. The modulated lights are collected by a lens and measured by a single-element detector, and subsequently converted to digital signals. Furthermore, the dedicated computer reconstructs the image of the target object from the light intensities. The FPGA part reconstructs an image, whereas the embedded CPU on the SoC FPGA generates the drawing on a display and initializes.

Figure 1 shows the schematic of the experimental setup. The experimental setup comprises of a camera lens (Thorlabs MVL50M23), white light-emitting diode (LED), condenser lens, digital micromirror device (DMD) (Vialux Hi–Speed V–Modules V7000), single-element detector (Thorlabs PDA100A2), analog-digital converter (Digilent Inc. Pmod AD1), and the dedicated computer. The light transmitted through the object is formed onto the DMD by the camera lens.

Real-time single pixel imaging is difficult because it requires sequentially the pattern display control, analog-digital converter control, and reconstruction calculation processing, but the proposed system can perform a real-time reconstruction of target objects by performing parallel processing. The reconstructed image is displayed on a display panel.

The experimental procedure of the real-time display is as follows. The object light is formed on the DMD by the camera lens. An encoding mask pattern is displayed on the DMD, and the object light is modulated by the pattern. The modulated light is collected by the lens and measured as a light intensity by the single-element detector. The obtained light intensity is converted to a digital signal from the analog intensity signal by the analog-digital converter. This operation is repeated while switching the encoding mask patterns. The receiving circuit in the FPGA saves converted signal in an FPGA built-in memory at the timing of asserting the synchronized signal, which is generated when the DMD switches to new encoding mask patterns. After the receiving circuit saves the signal a specified number of times, the reconstruction circuit starts calculating the target object image. Then, the embedded CPU on the SoC FPGA chip receives the reconstruction results and displays them on the display panel. Thus, we can observe the movie of the target object in real-time on the display panel by repeating the above procedure.

### Details of the dedicated computer

**Figure 2:**
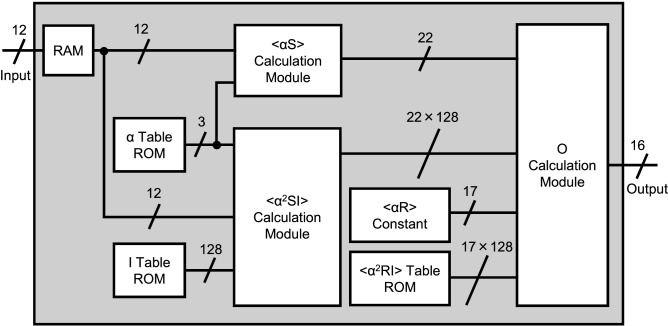
Block diagram of the reconstruction circuit. The number of encoding mask patterns can be up to 1024. The numbers above diagonal lines indicate the bit width of a signal. The reconstruction circuit comprises three calculation modules and lookup tables. We implemented 128 modules for $\langle \alpha ^2 SI \rangle$ calculation and *O* calculation for parallel processing.

We developed the dedicated computer using Zynq UltraScale+ MPSoC ZCU104 evaluation board provided by Xilinx. The schematic of the dedicated computer is shown in Fig. 1. We designed the circuit of the FPGA part using Vivado (version 2020.1) and the embedded CPU part using Vivado (version 2019.1), which are the integrated development environment for hardware design provided by Xilinx. The embedded CPU on the SoC FPGA runs a Linux operating system (OS) with the Linux kernel built using petalinux (version 2019.1) provided by Xilinx.

Figure 2 shows a block diagram of the reconstruction circuit. The reconstruction circuit performs the reconstruction calculation^25^ as in Eq. (1).

1 $$\begin{aligned} O(x,y) = \langle \alpha _i R_i \rangle \langle S_i {\alpha }^2_i I_i (x,y) \rangle - \langle \alpha _i S_i \rangle \langle R_i {\alpha }^2_i I_i (x,y) \rangle , \end{aligned}$$

where $I_i(x,y)$ is the light distribution of encoding mask patterns with binary values, the subscript *i* is an index of the encoding mask patterns; and $R_i = \sum _{y=1}^Y \sum _{x=1}^X I_i (x,y)$ is the total light intensity of the encoding mask patterns, and *X* and *Y* are the horizontal and vertical sizes of the encoding mask patterns. These values are only determined by the encoding mask patterns. $\langle \alpha _i \rangle = \frac{1}{N} \Sigma ^{N}_{i=1} \alpha _i$ is an ensemble average, where *N* is the number of the encoding mask patterns; $S_i = \sum _{y=1}^Y \sum _{x=1}^X T(x,y) I_i (x,y)$ is the light intensity measured by the single-element detector, where *T*(*x*, *y*) is the light distribution of the target object; and *O*(*x*, *y*) is the intensity distribution of a reconstructed target object. An image of the target object to be displayed on a display panel is obtained by normalizing *O*(*x*, *y*). In Eq. (1), the calculations not including $S_i$ can be precomputed, and the circuit must only compute the calculations including $S_i$. Although, the difference between the conventional and our reconstruction calculations is to introduce both encoding mask patterns optimization and the scaling factor $\alpha _i$, the image quality can be significantly improved with only a few additional operations. Essentially, the proposed method is suitable for FPGA implementation. Identification of optimal encoding mask patterns and $\alpha _i$ is explained in the “Methods”.

The reconstruction circuit receives $S_i$ as inputs and outputs *O*(*x*, *y*) as the calculation results. The input is received from the receiving circuit shown in Fig. 1, and the output is sent to the embedded CPU on the SoC FPGA. In this study, we developed two circuits for 512 and 1024 encoding mask patterns. The values determined by the encoding mask patterns are stored on lookup tables implemented by read only memories (ROMs) in the FPGA. The $\langle \alpha S \rangle$ calculation module, $\langle \alpha ^2 SI \rangle$ calculation module, and *O* calculation module perform the corresponding calculations in Eq. (1). All the operations are implemented by fixed-point number calculations as they require fewer hardware resources and are faster. We implemented 128 modules for $\langle \alpha ^2 SI \rangle$ calculations and 128 modules for *O* calculations; therefore, the dedicated computer can calculate 128 pixels in parallel.

### Simulation

**Figure 3:**
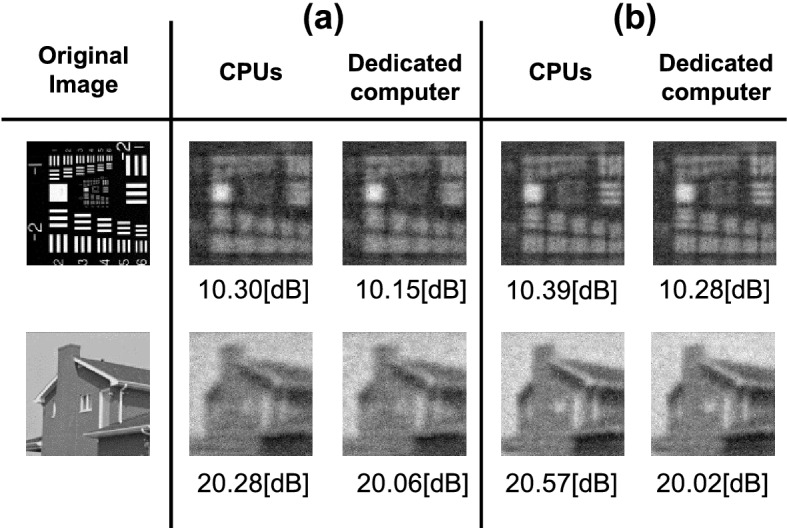
Simulation results of the desktop, embedded CPUs, and the dedicated computer. The size of the images is 128 × 128 pixels. We used single-precision floating-point operations on the CPUs and fixed-point operations on the dedicated computer. The number of the encoding mask patterns used in the reconstruction is 512 for (**a**) and 1024 for (**b**).

**Table 1:** Reconstruction times of each computer, and speed-up ratio relative to the embedded CPU alone.

Computer	512 patterns		1024 patterns	
	Processing time (ms)	Speed-up ratio	Processing time (ms)	Speed-up ratio
Embedded CPU alone	186.05	1.00	372.03	1.00
Desktop CPU	18.64	9.98	37.80	9.84
Dedicated computer	3.41	54.56	3.74	99.47

We evaluate the image quality and calculation speed of the reconstruction circuit in simulations and optical experiments. In addition, we show that the dedicated computer can reconstruct dynamic scenes in real-time using the experimental system.

Because the reconstruction circuit uses fixed-point operations, the image quality depends significantly on the bit width of each signal. The evaluation of the image quality is shown in Fig. 3. We used peak signal-to-noise ratio (PSNR) as image quality index. The reconstructed images using the desktop, embedded CPUs, and dedicated computer are similar. From these results, we confirmed that the dedicated computer could reconstruct the same image quality as the embedded and desktop CPUs.

We compared pure reconstruction times, not including pattern modulation, and analog-to-digital conversion times in the embedded CPU, desktop CPU, and dedicated computer. The results are shown in Table 1. The computing environment of the embedded CPU on the SoC FPGA is as follows: the CPU is Quad Core Arm Cortex-A53 MPCore (1.2 GHz), memory is 2.0 GB, the OS is Ubuntu 18.04.1 LTS, and compiler is gcc 7.5.0 (optimization option -O2 activated). The computing environment of the desktop CPU is as follows: the CPU is Intel® Core™ i5-4690 (3.5 GHz), memory is 32.0 GB, the OS is Windows 10 Enterprise, and compiler is visual studio 2015 (optimization option-O2 and streaming SIMD extensions activated). For the environment of the FPGA part, the operating frequency is 0.2 GHz. Each processing time are averaged 1000 measurement. From Table 1, it is evident that the dedicated computer is the fastest.

### Experimental results

**Figure 4:**
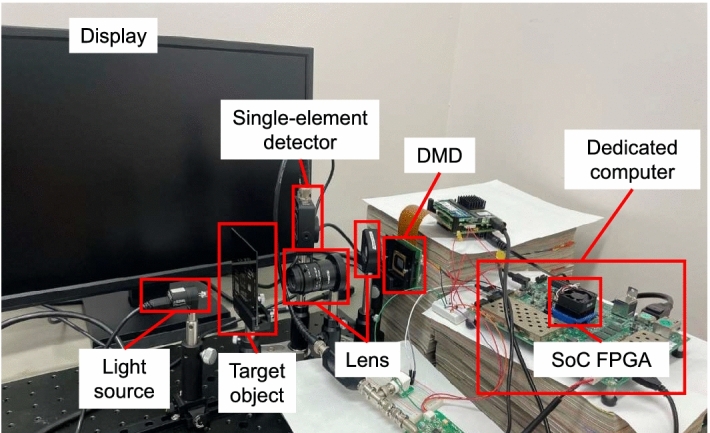
Optical system used in the experiment. The configuration and arrangement of the optical system is the same as in Fig. 1.

**Table 2:** Frame rate of each computer, and speed-up ratio relative to the embedded CPU alone.

Computer	512 patterns		1024 patterns	
	Frame rate (fps)	Speed-up ratio	Frame rate (fps)	Speed-up ratio
Embedded CPU alone	5.08	1.00	2.61	1.00
Desktop CPU	21.63	4.26	11.06	4.24
The dedicated computer	45.36	8.93	21.89	8.39

In the desktop PC, we used an analog-digital converter (ELMOS, FAD-16HS).

Experiments was conducted on a real-time display using our dedicated computer under the same conditions as the simulations. Figure 4 shows the optical system with the same configuration as Fig. 1. Table 2 shows the frame rate, which is defined as the time of displaying a previous reconstruction image to the start of displaying a next reconstruction image. The time required for displaying encoding mask patterns is 22.53 ms in 512 encoding mask patterns, and 45.06 ms in 1024 encoding mask patterns. In the real-time display experiment, we confirmed that the dedicated computer is the fastest.

**Figure 5:**
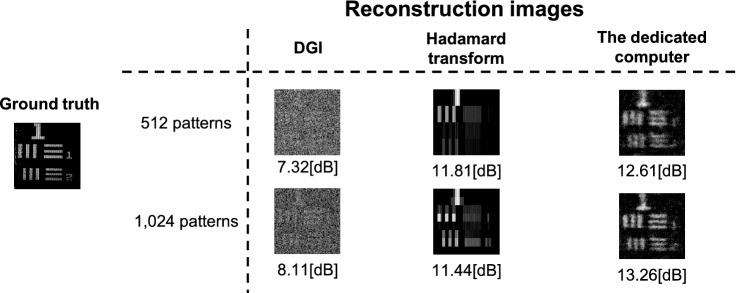
Reconstructed images by the dedicated computer. The image quality was evaluated by comparing with the ground truth reconstructed with the full sampling Hadamard transform. The image size is 128 × 128 pixels. Numbers of the encoding mask patterns are 512 and 1024, respectively.

**Figure 6:**
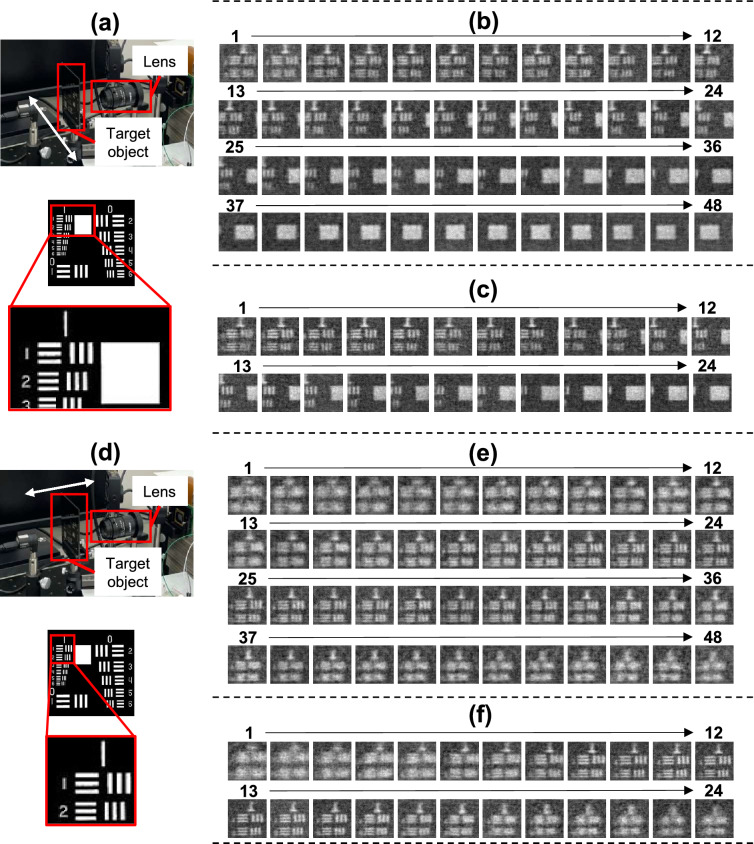
A reconstructed dynamic scene: (**a**) a target object (USAF1951 test target, Thorlabs R3L3S1N). The white arrow indicates the direction in which the target object is to be moved. The target object within the red rectangle moves in the direction of the arrow for 1 s, (**b**) reconstructed dynamic scene while moving the target object in the lateral direction in the case for 512 encoding mask patterns, (**c**) reconstructed dynamic scene while moving the target object in the lateral direction in the case for 1024 encoding mask patterns, (**d**) a target object when the movement direction is in the depth direction. The white arrow indicates the direction in which the target object is to be moved. The area indicated by the red rectangle was reconstructed. the target object was moved from $-1$ cm to $+1$ cm in 1 s, (**e**) reconstructed dynamic scene while moving the target object in the depth direction for 512 encoding mask patterns, (**f**) reconstructed dynamic scene while moving the target object in the depth direction for 1024 encoding mask patterns. The number of the reconstructed images is different since frames rate are different depending on the number of the encoding mask patterns, as shown in Table 2. The frame numbers are indicated above the images.

Figure 5 shows the evaluation of the reconstructed images by the dedicated computer. As a result, the reconstruction images obtained by the dedicated computer showed better image quality than previous FPGA-based studies^22,23^. Figure 6 shows the reconstructed dynamic scene using the dedicated computer. The reconstruction was performed in the cases of 512 and 1024 encoding mask patterns. Videos in the Supplementary Information show the real-time reconstruction movies using each computer in the cases of 512 and 1024 encoding mask patterns. Comparing these movies, we can see the dedicated computer can display the movement of a target objects smoothly. Noise of the embedded CPU alone and dedicated computer is significant compared to the desktop CPU due to the resolution of the analog-digital converter. The analog-digital converter of the desktop CPU has a 16-bit resolution, whereas the analog-digital converter of the embedded CPU alone and the dedicated computer has only a 12-bit resolution.

## Discussion

**Figure 7:**
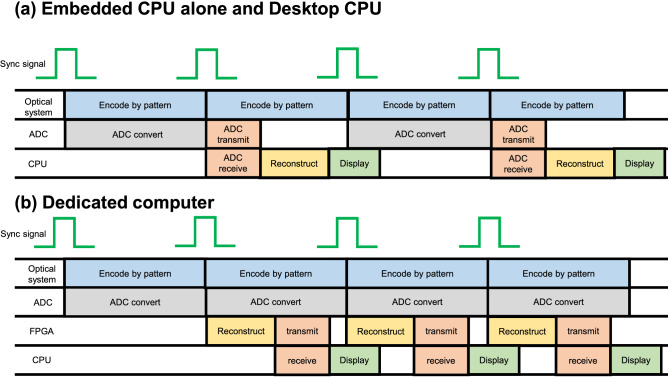
Time chart of the real-time display system. Each line shows the responsibilities of each process, executed in parallel. (**a**) Shows a time chart using the embedded CPU alone and desktop CPU. (**b**) Shows a time chart using the dedicated computer. The embedded CPU alone and desktop CPU perform processing to receive and transmit from the analog-digital converter because the analog-digital converter is controlled by the CPU. Thus, the embedded CPU alone and desktop CPU cannot perform analog-digital conversion constantly. In the embedded CPU alone, the reconstruction calculation takes a long time for encoding by patterns. In the desktop CPU, the reconstruction calculation time is short enough for the time of encoding by patterns. In contrast, the dedicated computer does not have such the time-consuming processing because the FPGA part receives a value of analog-digital conversion directly. Consequently, this allows the dedicated computer to perform analog-digital conversion constantly, thereby reducing the reconstruction calculation time.

Here, we have proposed the dedicated computer for single-pixel imaging using a SoC FPGA. A novelty of proposed dedicated computer is that parallelized reconstruction circuits are used in parallel based and a FPGA has been adopted with embedded CPU. In addition, the dedicated computer can perform task parallel processing including analog-digital conversions, reconstruction calculations, and display processing. The FPGA part in the Soc FPGA handles receiving measured intensities of the analog-digital conversion and the reconstruction calculation, and the embedded CPU part initialize the reconstruction circuit, and normalizes reconstruction results to 8-bit images, and displays the images on a display panel using OpenGL that open source software for computer vision. In particular, our dedicated computer can handle reconstructed images of 128 × 128 pixels that is larger size than previous studies using FPGAs^23,24^. To handle large images we must use ultraRAM, which has the largest capacity among built-in memories in the SoC FPGA. Since ultraRAM cannot be initialized from the FPGA part, it must be initialized by the embedded CPU when used as ROM.

We have analyzed the calculation speed of the dedicated computer. From Table 1, the calculation time of the embedded CPU alone and desktop CPU increase in proportion to the number of the encoding mask patterns. In contrast, the calculation time of the dedicated computer does not increase significantly as the number of encoding mask patterns increases. This is due to the communication time required in the dedicated computer occupies most of the entire processing time. The communication of the dedicated computer is only the transmission of reconstructed images to the embedded CPU since the encoding mask patterns are stored in the ROMs in the FPGA. Therefore, the communication time does not depend on the number of the encoding mask patterns. Thus, the dedicated computer becomes more advantageous in terms of the time required for reconstruction with the increase in the number of the encoding mask patterns.

From Table 2, it is evident that the dedicated computer is the fastest in the experiments. The reconstruction calculation is performed in parallel with displaying encoding mask patterns as in Fig. 7. The embedded CPU alone is slow since the reconstruction calculation is slower than displaying encoding mask patterns. In contrast, the desktop CPU and the dedicated computer are fast since the reconstruction calculation is faster than displaying encoding mask patterns.

The analog-digital conversion of the dedicated computer is faster than that of the desktop CPU. As shown in Fig. 7, in the desktop CPU, the analog-digital converter is controlled and communicated by software, whereas in the dedicated computer, the values of the analog-digital conversion are directly read by the FPGA, thereby resulting in higher throughput in the dedicated computer than desktop CPU. If the image size increases, the desktop CPU will be difficult to overlap the reconstruction calculation and displaying the encoding mask patterns, whereas the dedicated computer can readily overlap it, further increasing its usefulness.

## Methods

### Ghost imaging with encoding mask patterns optimization

**Figure 8:**
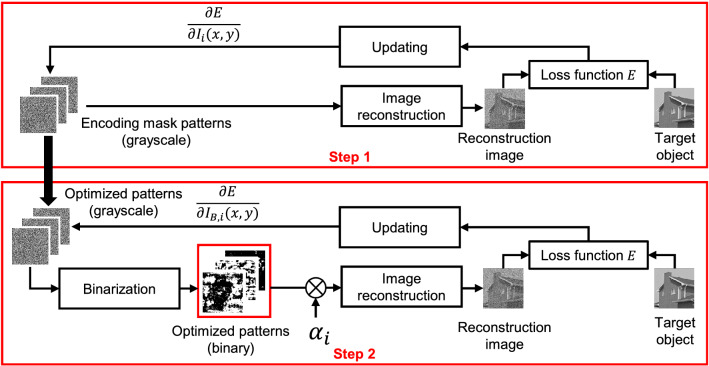
Procedure of the patterns optimization. In step 1, “Image reconstruction” calculates Eq. (1) without $\alpha _i$. “Loss function” *E* is a mean squared error. “Updating” performs gradient descent. “Binarization” performs ${I}_{B, i}(x,y) = 1$ if ${I}_{i}(x,y) \ge 0.5$ otherwise ${I}_{B, i}(x,y) = 0$.

**Figure 9:**
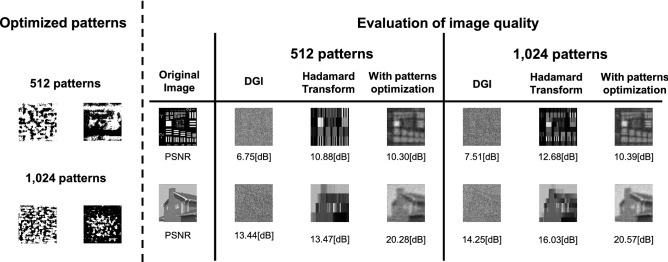
Comparison between the pattern optimization and conventional methods: An example of an optimized pattern is shown on the left side of the figure. The right side of the figure shows a comparison of reconstructed image quality. Image quality was compared with DGI^23^ and Hadamard transform^22^ implemented in an FPGA in previous studies. The size of the images was 128 × 128 pixels. The number of the encoding mask patterns were 512 and 1,024, respectively.

In the ghost imaging method^25^, optimized encoding mask patterns and scaling factor $\alpha _i$ in Eq. (1) were introduced to conventional DGI^31,32^. The optimized encoding mask patterns are obtained gradient descent, often used in machine learning. The scaling factor $\alpha _i$ in Eq. (1) is obtained as

2 $$\begin{aligned} \alpha _i = \mathop {\mathrm {arg~min}}\limits _{\alpha _i} \sum _{x=1}^{X} \sum _{y=1}^{Y} (I_{i}(x,y) - \alpha _i I_{B, i}(x,y))^2, \end{aligned}$$

where $$\alpha _i = \frac{\sum _{x=1}^{X} \sum _{y=1}^{Y}I_i (x,y) I_{B,i} (x,y)}{\sum _{x=1}^{X} \sum _{y=1}^{Y} I_{B,i}^2(x,y)}$$, $$I_{i}(x,y)$$ denotes grayscale encoding mask patterns before binarization; and $$I_{B, i}(x,y)$$ denotes binarized encoding mask patterns. The binarization is performed as $$ {I}_{B, i}(x,y) = 1 $$ if $$ {I}_{i}(x,y) \ge 0.5 $$ otherwise $$ {I}_{B, i}(x,y) = 0$$. Figure 8 shows the procedures of the pattern optimization. The optimization is performed in two steps. In step 1, we obtain optimized grayscale patterns. In step 2, we reoptimize grayscale patterns while binarizing the grayscale pattens already obtained in the step 1. Next, the image reconstruction is performed by using Eq. (1) with the binarized pattern, and grayscale patterns are updated by a loss function for which we used a mean squared error. By repeating the above procedure, we obtain final optimized binary patterns.

To compare our method with conventional methods, Fig. 9 shows an example of mask patterns and corresponding reconstructions using these methods. The DGI and Hadamard transform implemented in FPGA^22,23^ are compared as conventional methods. As can be seen in Fig. 9, the image quality with our encoding mask patterns optimization is drastically improved than that with the conventional methods.

## Supplementary Information

Supplementary Legends.

Supplementary Video 1.

Supplementary Video 2.

Supplementary Video 3.

Supplementary Video 4.

## Data Availability

The datasets used and/or analysed during the current study available from the corresponding author on reasonable request.
