# Supplementary material for: Real-time single-pixel imaging using a system on a chip field-programmable gate array
Source: Sci Rep. 2022 Aug 18;12:14097. doi: 10.1038/s41598-022-18187-8 (PMC9388629; doi:10.1038/s41598-022-18187-8)
Supplement: Supplementary file 1 — Supplementary Legends. [file 41598_2022_18187_MOESM1_ESM.pdf]

Movie1 caption:

A view of the real-time display with 512 encoding mask patterns

Movie2 caption:

A view of the real-time display with 1,024 encoding mask patterns

Movie3 caption and legend:

Comparison of the real-time reconstruction movies using each computer in the cases of 512 encoding mask patterns, (Embedded CPU alone) Video calculated using only embedded CPU alone, (Desktop CPU) Video calculated using Desktop CPU, (The dedicated computer) Video calculated using the dedicated computer, (Moving the target object) View of moving the target object.

Movie4 caption and legend:

Comparison of the real-time reconstruction movies using each computer in the cases of 1,024 encoding mask patterns, (Embedded CPU alone) Video calculated using only embedded CPU alone, (Desktop CPU) Video calculated using Desktop CPU, (The dedicated computer) Video calculated using the dedicated computer, (Moving the target object) View of moving the target object.
